# Clinical development of metabolic inhibitors for oncology

**DOI:** 10.1172/JCI148550

**Published:** 2022-01-04

**Authors:** Kathryn M. Lemberg, Sadakatali S. Gori, Takashi Tsukamoto, Rana Rais, Barbara S. Slusher

**Affiliations:** 1 Johns Hopkins Drug Discovery,; 2 Department of Oncology and The Sidney Kimmel Comprehensive Cancer Center,; 3 Department of Neurology,; 4 Department of Pharmacology and Molecular Sciences,; 5 Department of Medicine, and; 6 Department of Psychiatry and Behavioral Science, Johns Hopkins University School of Medicine, Baltimore, Maryland, USA.

## Abstract

Metabolic inhibitors have been used in oncology for decades, dating back to antimetabolites developed in the 1940s. In the past 25 years, there has been increased recognition of metabolic derangements in tumor cells leading to a resurgence of interest in targeting metabolism. More recently there has been recognition that drugs targeting tumor metabolism also affect the often acidic, hypoxic, immunosuppressive tumor microenvironment (TME) and non-tumor cell populations within it, including immune cells. Here we review small-molecule metabolic inhibitors currently in clinical development for oncology applications. For each agent, we evaluate the preclinical studies demonstrating antitumor and TME effects and review ongoing clinical trials. The goal of this Review is to provide an overview of the landscape of metabolic inhibitors in clinical development for oncology.

## Introduction

Tumor metabolism has been an area of basic science and clinical interest for many decades, dating to Otto Warburg’s classic observations of high tumor glycolytic rates in the presence of oxygen (1). Metabolic demands of leukemia cells for purine and pyrimidine base production were first targeted with the development of aminopterin (2) and later methotrexate as some of the earliest antimetabolite therapies. In more recent years it has become widely accepted that reprogrammed energy metabolism of some tumor cells improves their ability to generate substrates needed for biomass (e.g., reducing equivalents, nucleotides) (3). Considerable efforts have also been made to understand the relationship between mutations in oncogenes/tumor suppressors and the resulting changes to metabolic pathways (4). Recent examples exist of successful translation of discovered cancer cell metabolic derangements into FDA-approved metabolic inhibitors. For example, d-2-hydroxyglutarate–producing (D2HG-producing) mutations in isocitrate dehydrogenase (IDH) were identified in sequencing efforts of gliomas and acute myeloid leukemia (AML) (5, 6). Detailed understanding of the metabolic and epigenetic consequences led to development and approval of ivosidenib and enasidenib for relapsed/refractory IDH-mutated AML (7). However, for many other observed metabolic derangements in tumor cells, clinical translation has been more challenging. Reasons for this hurdle include the flexibility of cancer metabolic pathways to circumvent points of inhibition, leading to insufficiency of monotherapy; overlap with metabolism of healthy cells, which narrows the therapeutic index; and the difficulty of accessing some tumors of interest — particularly CNS tumors or tumors within a dense environment of supporting cells.

**Figure 1:**
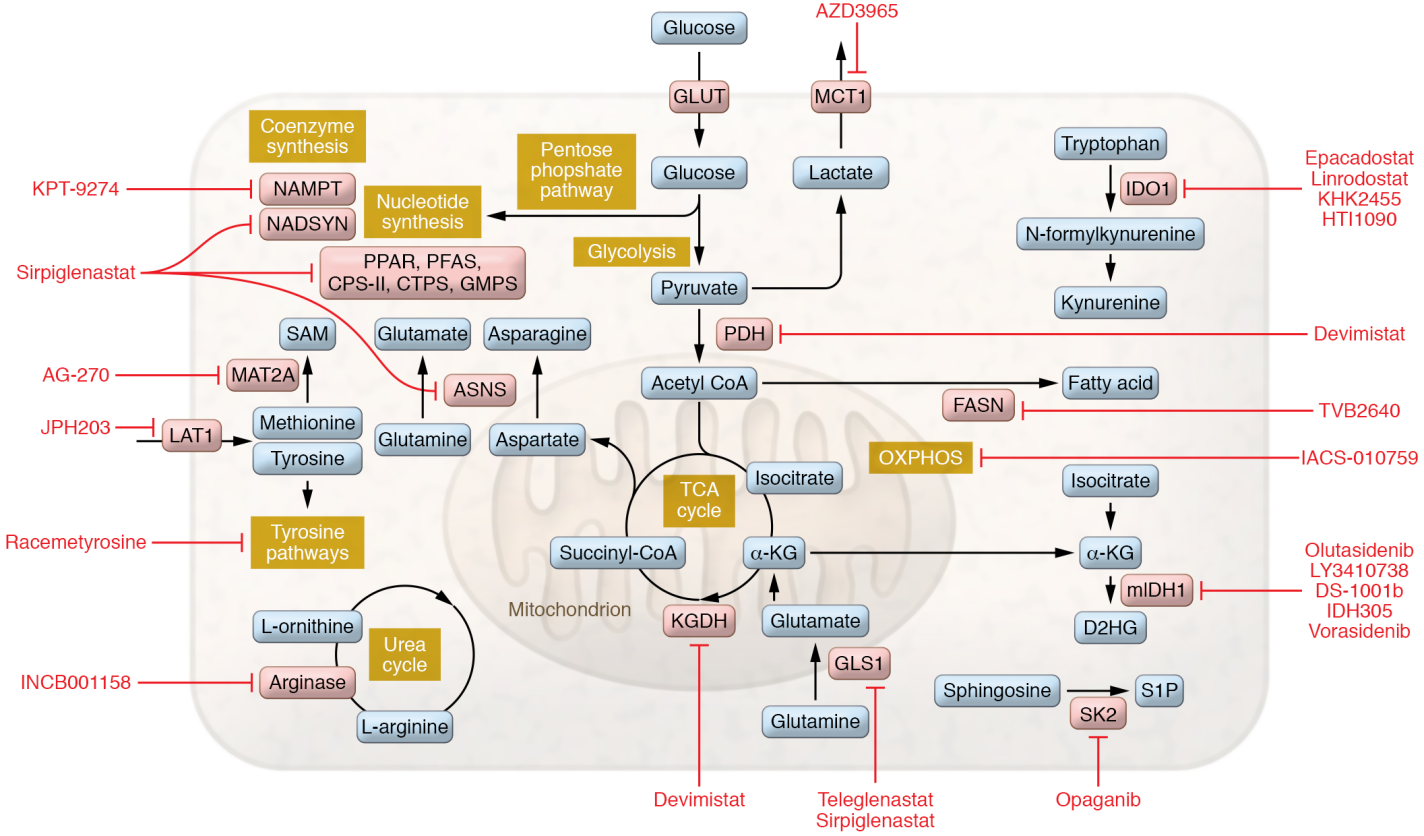
Metabolic inhibitors under clinical investigation for oncology applications. Schematic depicts the metabolic pathways and processes inhibited by agents described in this Review. Agents are shown in red text. Key enzymes are shown in pink. Pathways are labeled in yellow. Metabolites are shown in blue.

**Figure 2:**
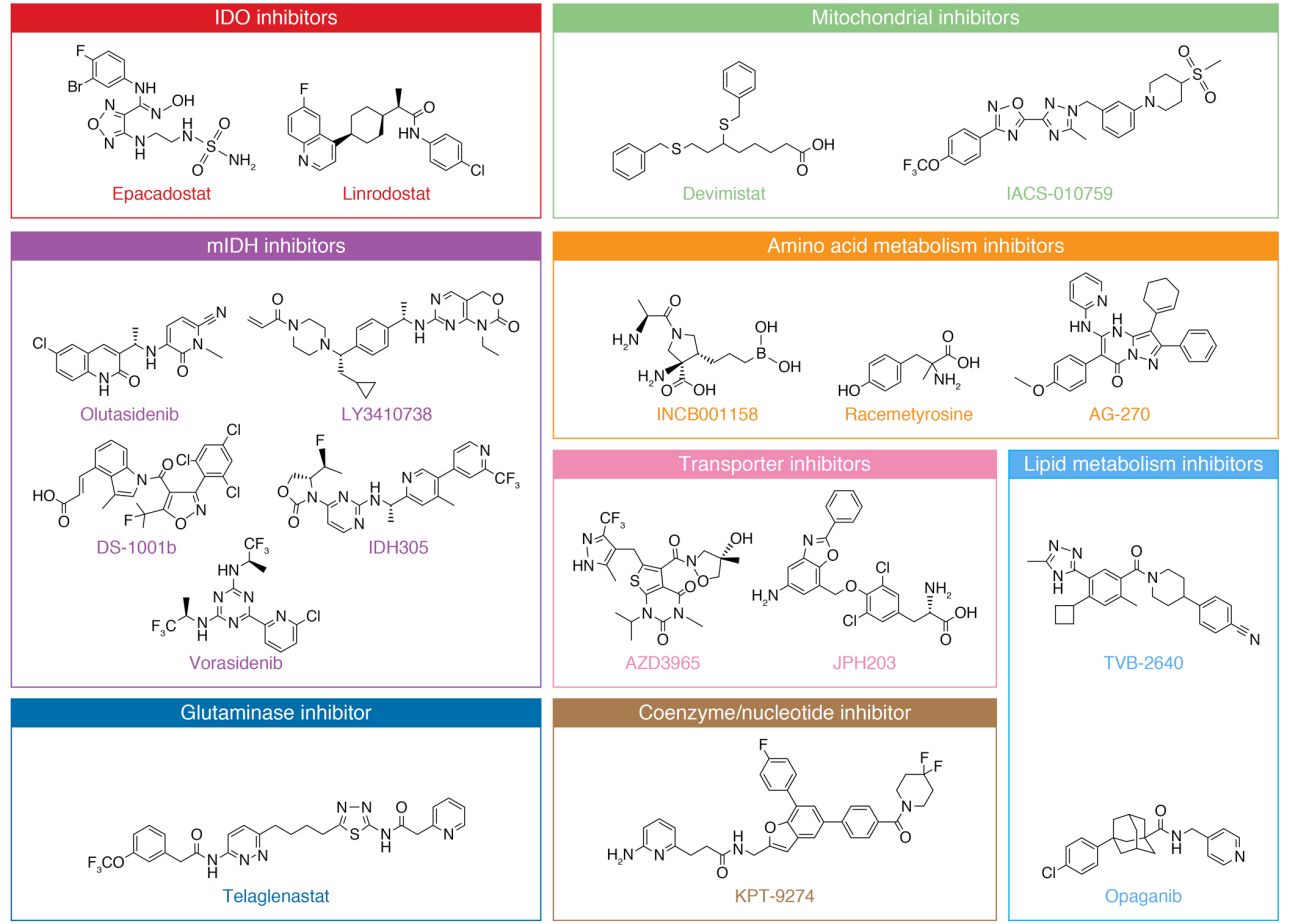
Structures of metabolic inhibitors currently under clinical investigation, grouped by target pathway. Structures of KHK2455, HTI-1090, and sirpiglenastat have not yet been disclosed.

The tumor microenvironment (TME) contains connective tissue, vessels, fibroblasts, and immune cell populations and plays a critical role in tumor growth, survival, and spread (4). Efforts have been made to target aspects of the TME in the treatment of cancer — for example with angiogenesis inhibitors (8), connective tissue antagonists (9), and immunomodulatory therapies. As the TME is often acidic, hypoxic, and nutritionally a suboptimal environment for immune surveillance and antitumor immune activity (10), there has been interest in characterizing the metabolic needs of antitumor immune cells and the effect of tumor metabolic inhibition on the greater TME. Several excellent recent reviews detail basic and early translational investigations into this area (11–13). Specifically, studies have focused on markers of immune susceptibility following metabolic inhibition and whether combination with immunotherapy may improve outcomes. In this Review we describe the current landscape of metabolic inhibitors in clinical development for oncology applications (Figure 1). These include agents with considerable clinical data (e.g., IDO1 inhibitors) as well as newer entries (e.g., sphingosine kinase-2 inhibitors). For each compound (Figure 2), we review available preclinical data (Table 1) and current clinical trials including novel combinations (Tables 2 and 3). The goal is to synthesize findings to date and motivate further translational research into the most effective ways to combine metabolic inhibition with chemotherapy and/or immunotherapy for improved treatment of cancer.

## Metabolic inhibitors in clinical trials

### Targeting IDO: second wave of clinical trials.

Indoleamine 2,3-dioxygenase 1 (IDO1) converts tryptophan, which is required for T cell activity, into the immunosuppressive metabolite kynurenine. Accumulating evidence suggests that many tumors and immune cells within the TME increase their IDO1 activity as part of a strategy to decrease T cell responses and elude anticancer surveillance (14, 15). The mechanisms by which IDO1 adversely affects the immune system are multifold and include inhibition of local CD8^+^ T effector (Teff) cells; increasing formation of CD4^+^ T regulatory cells (Tregs) (16) and myeloid-derived suppressor cells (MDSCs) (17); inhibition of mTOR; and activation of the aryl hydrocarbon receptor (18, 19). IDO1 can also be induced in specific subsets of antigen-presenting cells, leading to immune tolerance to tumor antigens (20).

Given the therapeutic potential, multiple small-molecule IDO1 inhibitors have been developed, and were shown to restore antitumoral T cell immunity and synergize with immune checkpoint inhibitors in preclinical models (21). Based on these encouraging results, several companies took IDO1 inhibitors into clinical development (22). Although initial phase I/II trials encouraged the idea that IDO1 inhibition may improve responses to anti–PD-1 immune checkpoint therapy, the failed randomized phase III studies of epacadostat and navoximod in metastatic melanoma in combination with pembrolizumab led to an abrupt pause in IDO1 inhibitor development. Postmortem cautionary narratives followed, arguing that the initial failures may have been due to inadequately planned clinical trials designed with limited preclinical data, lack of confirmed target engagement, lack of a rationale for patient selection, need for improvements to patient stratification, and/or lack of biomarkers to guide dosing (23). Thus, enthusiasm for IDO1 inhibitors was not extinguished, and with revised clinical strategies, development of several inhibitors was restarted across a range of tumor types, including metastatic prostate (24), bladder (25, 26), glioblastoma (27), endometrial (28), hepatocellular (29), and head and neck squamous cell carcinoma (30), with most studies including combinations with immunotherapy.

There also have been new entrants into the IDO1 field, including KHK2455, which is being evaluated in advanced bladder cancer (31) in combination with anti–PD-L1. Some of the newer inhibitors, such as linrodostat and KHK2455, are reported to be different from earlier inhibitors as they inhibit IDO1 by competing with heme for binding to the apoenzyme (apo-IDO1) (32). This binding event prevents apo-IDO1 from forming an active complex, ensuring a durable inhibitory effect (33). Targeting strategies that account for both IDO1 and tryptophan 2,3-dioxygenase (TDO) have also been rationalized, and dual IDO1/TDO inhibitors (e.g., HTI-1090/SHR9146) were in clinical trials (34), though the evidence for this approach to date is indirect (35).

Different drug combinations with IDO1 inhibitors are also being attempted. While there is a mechanistic rationale for the combination with anti–PD-1, more efficacious combinations have been demonstrated with DNA-damaging chemotherapies (36) and cancer vaccines (37). With better rationalized compounds and improved trial designs, the verdict is still out on the utility of this class of immune-modulating drugs.

### Targeting mutant IDH in the brain.

Isocitrate dehydrogenases 1 and 2 (IDH1 and IDH2) catalyze the oxidative decarboxylation of isocitrate to α-ketoglutarate (α-KG) in an NADP-dependent manner. These enzymes represent the most frequently mutated metabolic genes in human cancer (38). Mutations in IDH1 (at Arg132) and IDH2 (at Arg172) result in loss of the native catalytic activity but gain of a new catalytic function, namely, NADPH-dependent reduction of α-KG to D2HG (5, 39). Elevated D2HG can induce epigenetic alterations thought to play a critical role in malignant progression (40). Furthermore, D2HG was reported to suppress activation of the classical and alternative complement pathways as well as T cell response, potentially promoting immune escape of IDH-mutant tumors (41). These carcinogenic features of D2HG make mutant forms of IDH attractive targets for therapeutic agents specific to IDH-mutant cancers. Ivosidenib (AG-120) and enasidenib (AG-221) are first-in-class inhibitors of mutant IDH1 and IDH2 (mIDH1 and mIDH2) (42, 43), respectively, which were recently approved by the FDA for the treatment of AML with IDH1 or IDH2 mutation.

Frequent IDH mutations in brain tumors have prompted interest in improving CNS penetration of mIDH inhibitors. Several second-generation brain-penetrant inhibitors have been developed, including LY3410738 (44), DS-1001b (45), olutasidenib (FT-2102; ref. 46), and vorasidenib (AG-881; ref. 47), and multiple trials are under way in patients with mIDH glioma (48–53). Because elevated D2HG can lead to genomic CpG hypermethylation, some of these inhibitors are being investigated in combination with DNA hypomethylating agents (54, 55). A recent preclinical study suggests that mIDH1 inhibition may improve survival when combined with anti–PD-L1 (56). Toward this end, olutasidenib combined with nivolumab is being evaluated in patients with advanced solid tumors and gliomas with mIDH1 (57).

### Inhibition of glutaminase by telaglenastat (CB-839).

Glutamine is a critical fuel for metabolic processes; it serves as a nitrogen and carbon source in biosynthetic pathways and is essential for energy generation as well as cellular homeostasis. Several cancers have been described as “glutamine-addicted” because of their exceptional metabolic demands.

Telaglenastat (CB-839) is a first-in-class, potent and selective inhibitor of the enzyme glutaminase (GLS1), which catalyzes the deamidation of glutamine to glutamate. Telaglenastat inhibits both splice variants of GLS1, the kidney type (KGA) and glutaminase C (GAC), with nanomolar potency (58). Glutaminolysis inhibition by telaglenastat has been shown to prime the tumor environment for immunological modulation, including enhancing the cytotoxic activity of tumor-infiltrating lymphocytes (TILs) (59, 60). Telaglenastat has shown efficacy in several preclinical cancer models that exhibit glutamine dependence, including triple-negative breast cancer (58), non–small cell lung cancer (NSCLC) (61), AML (62), renal cell carcinoma (RCC) (63, 64), and lymphoma models. Antiproliferative responses were shown to correlate with elevated GLS1 protein, particularly GAC (58, 65). These favorable data supported moving telaglenastat into clinical development.

Telaglenastat was well tolerated at daily doses greater than 1 g with some evidence of single-agent activity in relapsed/refractory leukemia patients (66). However, in general, the single-agent activity was modest, and subsequent efforts have focused on combination therapies. For example, a combination study with panitumumab and irinotecan is under way for treatment of metastatic and refractory RAS-wild-type colorectal cancer using PET/CT imaging biomarkers (67). Telaglenastat is also being investigated in metastatic RCC in combination with the multikinase inhibitor cabozantinib (68), as promising effects were observed preclinically and in a small expansion cohort of RCC patients in phase I trials (63). Early data from primary analysis of this trial show that the combination was well tolerated and the adverse events were consistent with known risks of both agents; however, the addition of telaglenastat did not improve the efficacy of cabozantinib in RCC. The median progression-free survival (PFS) was 9.2 months among patients treated with telaglenastat and cabozantinib, which was similar to the 9.3 months observed with cabozantinib alone (69). Other combinations include carfilzomib (protease inhibitor) and dexamethasone for treating recurrent multiple myeloma (70); niraparib or talazoparib [poly(ADP-ribose) polymerase (PARP) inhibitors] for treating platinum-resistant BRCA-wild-type ovarian cancer (71); palbociclib (cyclin-dependent kinase inhibitor) for treating solid tumors (72); and sapanisertib (mTOR inhibitor) for treating advanced NSCLC (73).

In addition to combination studies, telaglenastat is also being explored in patients with specific tumor mutations. A phase II basket trial is evaluating telaglenastat in patients with tumors bearing NF1, KEAP1/NRF2, and STK11/LKB1 aberrations (74). KRAS-mutant tumors with functional inactivation of LKB1 and a KEAP1 co-mutation exhibit increased glutamine dependence to successfully adapt to oxidative and energetic stress. This metabolic requirement for glutaminolysis presents a therapeutic vulnerability in cancers with genetic, epigenetic, or post-transcriptional alterations in the KEAP1/NRF2 pathway (75). Another early-stage trial is exploring combinations with radiation therapy and temozolomide for IDH-mutated astrocytomas (76). Trials of telaglenastat in combination with osimertinib (kinase inhibitor) for treating EGFR-mutated stage IV NSCLC (77) and in combination with pembrolizumab/standard-of-care chemotherapy in patients with KEAP1/NRF2–mutated NSCLC are also under way (78).

### Broad glutamine antagonism with sirpiglenastat (DRP-104).

Sirpiglenastat (DRP-104) is a tumor-targeted prodrug of the glutamine antagonist 6-diazo-5-oxo-l-norleucine (DON), which was identified in the 1950s as a potent anticancer agent (79). DON is a mechanism-based, irreversible inhibitor of all glutamine-utilizing enzymes (80), and thus broadly inhibits metabolic pathways that require glutamine as a nutrient source. The main impediment in the clinical development of DON has been its dose-limiting toxicities to normal tissues (81), especially the gastrointestinal tract, which is highly glutamine dependent (82). By utilizing promoieties that are preferentially cleaved by tumor-enriched enzymes (83), sirpiglenastat is able to deliver DON preferentially to the tumor, increasing its therapeutic index.

Recent pioneering studies using an earlier glutamine antagonist prodrug, JHU083, discovered divergent metabolic pathways in tumor cells versus immune cells within the TME. While glutamine antagonism blocked oxidative and glycolytic metabolism in tumor cells, it made the TME less hypoxic, acidic, and nutrient-deprived, leading to marked upregulation of oxidative metabolism in the T cells, resulting in enhanced antitumor activity (84). JHU083 was also shown to markedly inhibit the generation and recruitment of MDSCs and increase inflammatory tumor-associated macrophages (TAMs) (85). This disentangled process has also been noted with sirpiglenastat, which significantly inhibits tumor growth with a tandem increase in TILs (including T cells, NK cells, and TAMs) and decreased MDSCs in mice with syngeneic MC38 tumors. Sirpiglenastat was found to be superior to anti–PD-1/PD-L1 monotherapy and showed antitumor synergy as a combination, resulting in long-term durable cures (86, 87). Sirpiglenastat also demonstrated promising effects in a glutamine-dependent KRAS-mutant lung cancer model that carries KEAP1 mutations (88, 89) and reduced KEAP1-mutant tumor growth in both murine and patient-derived lung and squamous tumor models (88). These data suggest that sirpiglenastat may be a promising therapeutic agent in patients carrying such mutations.

Sirpiglenastat is currently being evaluated in phase I/IIa clinical trials as a single agent and in combination with atezolizumab for the treatment of advanced solid tumors (90). Recently, sirpiglenastat received a fast-track designation for the treatment of advanced NSCLC patients whose tumors express mutations in KEAP1, NFE2L2, and/or STK11. A phase Ib trial in NSCLC patients is also planned to establish safety and tolerability in combination with pembrolizumab.

### Lactate efflux inhibition with AZD3965.

AZD3965 is a first-in-class, potent (*K_I_* = 1.6 nM), orally bioavailable, selective inhibitor of monocarboxylate transporter 1 (MCT1). As tumor cells show an increased dependence on glycolysis resulting in lactic acid production, they overexpress MCTs as a protective mechanism to efflux lactate and avoid intracellular acidification. Potent antiproliferative activity of AZD3965 has been demonstrated in multiple lymphoma cell lines (91) and in small cell lung cancer (SCLC) models (92). A pharmacodynamic biomarker study showed that AZD3965 therapy resulted in decreased lipid synthesis including decreased choline levels. The drop in choline was attributed to decreased choline kinase A levels following intracellular lactate accumulation (93). Studies also showed that AZD3965-treated tumors had an increased abundance of dendritic cells and NK cells. Another publication assessed proteomic perturbation in mammospheres isolated from ER-positive breast cancer cell lines (94) and found dramatic overexpression of mitochondrial proteins, implying that cancer stem cells may become resistant to stress by fortifying the capacity to produce ATP via oxidative mitochondrial metabolism. Currently AZD3965 is being evaluated in a phase I dose-ranging study (95). While limited data are available at this point, one published case report noted malignant hyperlactemic acidosis in a patient with metastatic melanoma following the first dose of AZD3965 (96). The patient, in a PET scan, showed high glucose uptake in the metastasized tumors with extensive disease burden while minimal uptake was observed in the brain, suggesting a Warburg effect–dominated lactate production and efflux from the tumors. However, off-target effects of MCT inhibition in key tissues such as liver and kidney by AZD3965 treatment presumably interfered with plasma clearance of lactate, thus precipitating the symptomatic deterioration.

### Inhibition of mitochondrial metabolism with devimistat (CPI-613) and IACS-010759.

While appreciation of the Warburg effect has focused much of cancer metabolism research on aerobic glycolysis, mitochondrial metabolism has also been explored to target cancer. While efforts have generally focused on tumor cells, certain populations of immune cells, such as Tmem, Teff, and Treg cells, are also thought to rely on mitochondrial respiration and oxidative phosphorylation (OXPHOS) in the hypoxic TME (11, 97–99), but there have been limited reports on this activity with the current clinical agents, representing an area for exploration.

The mitochondrial inhibitor devimistat (CPI-613) is a lipoate analog that inhibits two lipoate-dependent enzymes, ketoglutarate dehydrogenase (KGDH) and pyruvate dehydrogenase (PDH), that control availability of substrates derived from glucose and glutamine for replenishing the citric acid cycle (100, 101). The mechanism of inhibition of these enzymes appears to be distinct: devimistat was shown to modulate KGDH in a redox-dependent manner (102), whereas phosphorylation is affected for PDH (100). Preclinical studies using devimistat showed single-agent activity in xenograft models for NSCLC and pancreatic tumors (100) and activity in combination with chloroquine in clear cell sarcoma (103). However, limited evidence exists from preclinical studies showing an effect of devimistat on the TME or specific immune cell populations. One recent study from a gastroadenocarcinoma model highlighted improved survival in deinnervated gastric cancer treated with devimistat in combination with the mTOR inhibitor RAD001, highlighting a possible role for nervous tissue in the TME of these tumors (104). As a single agent, devimistat has been evaluated in multiple phase I studies, including in combination with modified FOLFIRINOX (mFOLFIRINOX) for pancreatic cancer (105, 106) and in combination with cytarabine/mitoxantrone for relapsed/refractory AML. In pancreatic cancer, devimistat plus mFOLFIRINOX exhibited a 61% overall response rate (ORR) (105). In the AML study, there was an ORR of 50% and the drug appeared promising in older patients and those with poor risk cytogenetics (107). Pretreatment bone marrow samples from a subgroup of AML patient responders showed an increased B lymphocyte gene signature, suggesting a role for B cells in the activity of the combined treatment. Further work will be needed to investigate this observation and identify whether a potential clinical role for devimistat in combination with immune checkpoint inhibitors exists. Current phase II/III clinical trials of devimistat include combinations with mFOLFIRINOX (108) in metastatic pancreatic cancer and with cytarabine/mitoxantrone in older adults with relapsed/refractory AML (109).

A mitochondrial complex I inhibitor, IACS-010759, is also in clinical oncology trials (110). IACS-010759 inhibits growth and induces apoptotic cell death in tumor models thought to be reliant on OXPHOS. In AML cells, metabolic changes following IACS-010759 treatment were thought to be due to decreased availability of substrates to fuel nucleotide biosynthesis (e.g., aspartate) (110). In a mouse model of melanoma brain metastases, IACS-010759 treatment decreased markers of tumor hypoxia (111). Consistent with this finding, in patient samples of melanoma brain metastases, gene expression data suggested suppression of immune cell genes and upregulation of OXPHOS genes, particularly in comparison with matched primary melanoma and lung metastases (111). In a PD-1–resistant NSCLC model, combination treatment with anti–PD-1 plus IACS-010759 improved treatment response compared with single agent (112). Human trials are in an early stage: partial results of a completed phase I study in advanced solid tumors have been reported (113), with an additional phase I trial ongoing in AML (114). Urine lactate is being explored as a biomarker of activity.

### Targeting of arginine metabolism with INCB001158 (CB-1158).

Several studies have investigated modulation of the amino acid arginine as an antitumor approach. Interactions between tumor cell arginine synthesis and pyrimidine metabolism have been shown to promote tumor growth (115, 116). Myeloid and Treg populations in the TME express high levels of arginase I, which can hydrolyze arginine and disrupt T cell expansion in the TME (117). The small molecule INCB001158 (CB-1158) was developed to inhibit arginase I and increase arginine availability. This agent was tested in a phase I/II clinical study in combination with gemcitabine for advanced biliary cancers (118) with median PFS 8.5 months (119). A recent study showed that myeloid cells expressing arginase I suppress T cell proliferation in vitro, and this could be reversed by INCB001158 (120). Tumor xenograft models treated with INCB001158 showed decreased growth and increased T cell infiltration (120). A phase I clinical trial of INCB001158 as a single agent and in combination with anti–PD-1 in patients with solid tumors is ongoing (121); preliminary data suggested that the orally available drug is well tolerated (122).

### Mimicking tyrosine with racemetyrosine (SM-88).

Racemetyrosine (SM-88) is a tyrosine mimetic being developed for the treatment of various cancers, including pancreatic, lung, breast, prostate, sarcoma, and lymphoma (123). Some cancer cells more avidly take up and consume tyrosine compared with normal cells. Racemetyrosine takes advantage of this difference and acts as a dysfunctional tyrosine to interrupt tyrosine-mediated metabolic pathways, including protein synthesis. Specifically, racemetyrosine is thought to disrupt synthesis of cancer cell mucin-1 protein, leading to increased oxidative stress (124), cell death, and enhanced immunogenicity. In some cell lines, the methyl ester of racemetyrosine (SM-88 ME) was found to increase ROS in a dose-dependent manner (125). In a mouse colon cancer xenograft model, racemetyrosine was reported to reduce HCT116 tumor growth (125). A first-in-human, open-label, pilot study of racemetyrosine was conducted in advanced metastatic cancer patients in combination with melanin, phenytoin, and sirolimus (SMK therapy) (123). In addition, a phase Ib/II, open-label, dose-escalation study of racemetyrosine was conducted in combination with methoxsalen, phenytoin, and sirolimus in patients with non-metastatic biochemically recurrent prostate cancer (126). Although the outcomes of these open-label studies appear encouraging (e.g., no serious adverse events) (127, 128), more definitive conclusions await placebo-controlled randomized clinical trials.

### Inhibition of LAT1 with JPH203.

JPH203 is a selective inhibitor of large neutral amino acid transporter 1 (LAT1), a heterodimeric transporter composed of a heavy subunit protein, 4F2hc, encoded by the *SLC3A2* gene, and a light subunit protein encoded by the *SLC7A5* gene (129). LAT1 preferentially transports branched-chain amino acids (valine, leucine, isoleucine) and aromatic amino acids (tryptophan, tyrosine). LAT1 is overexpressed in many cancer cells, which consume these amino acids to sustain proliferation. Indeed, it was reported that overall survival and PFS of RCC patients were shorter in patients with high LAT1 versus those with low LAT1 expression (130). Because slowly dividing normal cells can rely on LAT2 for amino acid uptake (131), selective inhibition of LAT1 by JPH203 is believed to be cytotoxic to cancer cells while sparing normal cells. JPH203 showed antitumor activity in preclinical models of colon cancer (132), cholangiocarcinoma (133), and thyroid carcinoma (133). Preclinical data suggest that LAT1 inhibition may also disrupt uptake of citrulline, a precursor of arginine, by activated T cells, resulting in immunosuppressive effects by arginine deprivation (134).

A first-in-human open-label phase I study of JPH203 was conducted in Japanese patients with advanced solid tumors (135); a partial response was reported in one patient. The study indicated that the safety and efficacy of JPH203 could be predicted by genetic variants in the *NAT2* gene, which encodes an *N*-acetyltransferase responsible for phase II metabolism of JPH203. The study also suggested that plasma free amino acid levels and BMI are useful predictors of JPH203 efficacy (136). Randomized controlled phase II studies in patients with advanced biliary tract cancer are under way in Japan (University Hospital Medical Information Network [UMIN] Clinical Trials Registry UMIN000034080).

### Inhibition of MAT2A with AG-270.

Efforts have been made to target methionine metabolism in tumors with deletion of methylthioadenosine phosphorylase (*MTAP*) (137), which is often co-deleted with the tumor suppressor CDKN2A. Deletion of *MTAP* results in accumulation of its substrate, methylthioadenosine, which has been shown to be an endogenous inhibitor of protein arginine methyltransferase 5 (PRMT5). The PRMT5-catalyzed enzymatic process can be further downregulated by starving the enzyme of its substrate, *S*-adenosyl methionine (SAM), via inhibition of methionine adenosyltransferase 2α (MAT2A). Because PRMT5 plays a critical role in the pathological progression of several human cancers, MAT2A inhibition is considered synthetically lethal with *MTAP* deletion (138).

AG-270 is an orally active inhibitor of MAT2A, which dose-dependently reduced SAM levels in tumor and blocked tumor growth in a pancreatic xenograft model (137). The preliminary results from an ongoing, first-in-human, phase I trial of AG-270 in patients with advanced solid tumors with homozygous deletion of *MTAP* showed reduction in plasma SAM across a range of doses, demonstrating target engagement (139). The next step in this phase I study is to evaluate AG-270 in combination with taxane-based chemotherapy, which demonstrated synergistic antiproliferative effects in preclinical MTAP-null tumor models (138).

### Inhibition of fatty acid synthesis with TVB-2640.

Fatty acid synthase (FAS), encoded by the *FASN* gene, possesses multiple catalytic domains, which participate in the biosynthesis of long-chain fatty acids, mainly palmitic acid, from acetyl-CoA and malonyl-CoA. Since FAS was identified as a marker of poor prognosis in breast cancer patients (140), it has gained considerable attention as a therapeutic target. TVB-2640 is the most advanced of the FAS inhibitors reported to date. Although limited information is available on TVB-2640 (141, 142), its close analog, TVB-3166, was found to induce apoptosis in tumor cells and inhibit xenograft tumor growth as monotherapy (143). It was also reported to enhance the antitumor activity of taxanes through inhibition of tubulin palmitoylation and disruption of microtubule organization (144). A first-in-human study of TVB-2640 alone and with a taxane in advanced tumors demonstrated a manageable safety profile and potent target engagement (FAS inhibition) (145). TVB-2640 is now being studied in several phase II trials, including in patients with first relapse of high-grade astrocytoma in combination with bevacizumab (146); in combination with paclitaxel and trastuzumab in patients with HER2-positive metastatic breast cancer resistant to trastuzumab and taxane-based therapy (147); and in patients with KRAS-mutant NSCLC (148). It was recently reported that FAS contributes to functional maturation of Tregs and that loss of FASN from Tregs inhibits tumor growth, suggesting the therapeutic potential of this class of inhibitors in cancer immunotherapy (149).

### Alteration of bioactive lipids with opaganib (ABC294640).

Lipid metabolism and the effect of bioactive lipids on tumor cells and the TME are an active area of research and therapeutics development. Opaganib (ABC294640) is a sphingosine kinase-2 (SK2) inhibitor that reduces formation of the pro-proliferative, prosurvival lipid sphingosine-1-phosphate (S1P) (150). Opaganib suppressed proliferation of multiple tumor cells in vitro and reduced tumor growth in mice with mammary adenocarcinoma xenografts. Given wide interest in sphingolipids in inflammation, there are multiple preclinical studies showing immune-modulating effects of opaganib, though not in oncology models. These include an LPS model of inflammation in which targeting SK2 with opaganib increased macrophage inflammatory cytokine production (151). In a phase I study, opaganib was found to decrease plasma S1P levels as a marker of target engagement (152). Phase II studies in cholangiocarcinoma in combination with hydroxychloroquine (153) and in metastatic castration-resistant prostate cancer (154) are ongoing.

### NAD salvage inhibition with KPT-9274.

Many cancer cells upregulate nicotinamide phosphoribosyltransferase (NAMPT), the rate-limiting step for NAD^+^ salvage; thus efforts have been made to target this pathway with NAMPT inhibitors (155). KPT-9274 is a dual p21-activated kinase 4 (PAK4)/NAMPT inhibitor (156). It reduces the NAD^+^/NADH ratio in tumor cells and inhibits tumor growth in mouse models of sarcomas (156) and RCC (157). Interestingly, in KPT-9274–treated rhabdomyosarcoma xenografts, tumors showed transcriptomic enrichment in pathways of adaptive immunity, IFN-α and -γ response, and antigen processing (156). Using another NAMPT inhibitor (GMX1778) targeted to murine glioblastoma multiforme by microparticles, investigators found increased markers of TILs and increased surface expression of PD-L1. In this model there was a robust survival advantage in animals treated with immune checkpoint inhibitor plus NAMPT inhibitor (158). A phase I study of KPT-9274 was recently completed (159), and a study of KPT-9274 in combination with nivolumab for melanoma was enrolling patients but recently terminated (160).

## Summary

The breadth of metabolic pathways being targeted in oncology with new clinical compounds is evidence of how much has been learned about tumor metabolism from basic and translational research studies over the past few decades. Several established targets have multiple clinical compounds in later stages of development across a variety of tumor types (e.g., IDO1, IDH1), while many earlier-phase studies are being initiated with first-in-class agents targeting metabolic nodes not yet explored (e.g., IACS-010759, opaganib). Considering the critical role played by various metabolic pathways in normal cellular homeostasis, one major challenge in developing metabolic inhibitors for oncology lies in achieving antitumor activity without substantial impact on non-cancer cells. Some of the investigational drugs described above have been developed using strategies to improve the therapeutic index. For instance, as mentioned earlier, inhibition of glutamine metabolism by DON is toxic to the gastrointestinal tract, which is enriched with highly glutamine-consuming cells (82). As demonstrated by sirpiglenastat, however, the prodrug approach exploited for tumor-targeted delivery of DON appears to be effective in minimizing gastric toxicity. Furthermore, inhibition of neomorphic functions of mutant enzymes offers a unique opportunity to develop anticancer agents with a greater therapeutic index as demonstrated by the selective inhibition of mIDH1 and mIDH2.

One of the advantages unique to metabolic inhibitors is that biomarkers measurable in plasma can be found among substrates and products of target enzymes. Indeed, available biomarkers played a crucial role in the development of many of the metabolic inhibitors reviewed in this article. Measurements of D2HG particularly in relation to serum drug pharmacokinetics were used in the development of IDH1 inhibitors (46). Tumor and plasma levels of SAM, a product of the MAT2A-catalyzed reaction of methionine and ATP, were used as biomarkers of AG-270 target engagement in mouse xenografts (133) and a first-in-human trial (139), respectively, providing guidance for dose selection in future clinical studies. Similarly, urine lactate is being used as a biomarker in the trial of IACS-010759 after being found to be elevated in preclinical studies (110). Finally, as described above, imaging biomarkers using nuclear medicine tracers have been incorporated into clinical trials of telaglenastat (161). Given the remarkable technological advances in recent years in the areas of bioanalysis and metabolic profiling, we expect metabolic biomarkers will play an even larger role in future development.

At the same time, a challenge for development of metabolic inhibitors in oncology is in identifying the patients whose tumors are most likely to benefit from a given agent. Lack of or incomplete understanding of biomarkers for patient enrollment has been cited as a contributing factor for the negative results in clinical studies of metabolic inhibitors, including the ECHO-301 trial combining IDO1 inhibitor with immunotherapy (23). On the other hand, several strategies to identify patients most likely to respond have been used successfully with other metabolic inhibitors. One strategy is to make use of genetic markers found in tumor cells. This is useful when an identified gene mutation can be associated with the metabolic target of interest; this strategy was used in trials of the IDH inhibitors ivosidenib and enasidenib (7). Deletion of *MTAP* is being used for patient selection in trials of AG-270 (ClinicalTrials.gov NCT03435250). For other metabolic inhibitors, such as the glutaminase inhibitor telaglenastat, surrogate genetic biomarkers leading to tumor glutamine dependence are being employed (e.g., NRF2/KEAP1) (75). A second potential patient selection strategy includes the use of imaging markers to evaluate tumor metabolic pathways and follow early response to therapy. As an example, hyperpolarized ^13^C-pyruvate MRI has been identified as a noninvasive way of evaluating prostate cancer, where conversion from ^13^C-pyruvate to ^13^C-lactate is measured and used to identify tumor tissue and treatment response with greater sensitivity than conventional ^1^H-MRI techniques (162, 163). Hyperpolarized ^13^C-pyruvate–to–lactate flux has been associated with higher MCT-expressing tumors, raising the possibility of pairing this companion imaging technology with clinical trials of MCT inhibitors. With additional probes and technology innovations in active development (164), hyperpolarized MRI and other forms of metabolic imaging will be useful additions for clinical oncology patient selection and follow-up in future trials.

An area of focus in this Review was to describe the effects of metabolic inhibitors on the TME in preclinical studies and understand how these findings were translated to clinical studies. For several of the agents highlighted, however, the effect on the TME was not well explored. Additionally, many published preclinical studies used nonclinical compounds, which can present a challenge in directly interpreting the results for a clinical agent. For example, olutasidenib is currently in clinical trials in combination with nivolumab for advanced solid tumors and glioma (57), but published literature on use of an IDH inhibitor with immunotherapy used a nonclinical tool inhibitor, which showed limited efficacy (56). It will be of interest to learn whether the preclinical data collected with clinical candidates are available when in-human study results are published. In addition, as preclinical oncology models improve to better reflect the TME of human tumors (e.g., humanized rodent models), we anticipate it will be more straightforward to investigate the in vivo effects of metabolic inhibitors on the TME preclinically.

It is worth noting that additional classes of metabolic agents in development exist outside the scope of this Review, including small-molecule receptor blockers with consequences for metabolic processes (e.g., trigluazole; ref. 165), antibody-based therapies (e.g., anti-CD73; ref. 166), enzyme-based metabolic inhibitors (e.g., ADI-PEG-20; ref. 167), and metabolic inhibitors already developed for other diseases now under investigation in cancer (e.g., eflornithine; ref. 168). Metabolic manipulations for ex vivo priming of immune cells directed at cancer are an additional fascinating area of research, which has been discussed elsewhere (169).

While multiple strong reviews in the literature cover the basic and translational processes leading to development of metabolic inhibitors, this Review offers a unique perspective by focusing on those agents that have progressed to human clinical studies in oncology. With significant drug development efforts in this area, it is likely that with the right combination with chemo- and/or immunotherapy, several of the metabolic inhibitors outlined in this Review will be widely employed in clinical oncology in the future.

## Figures and Tables

**Table 3:**
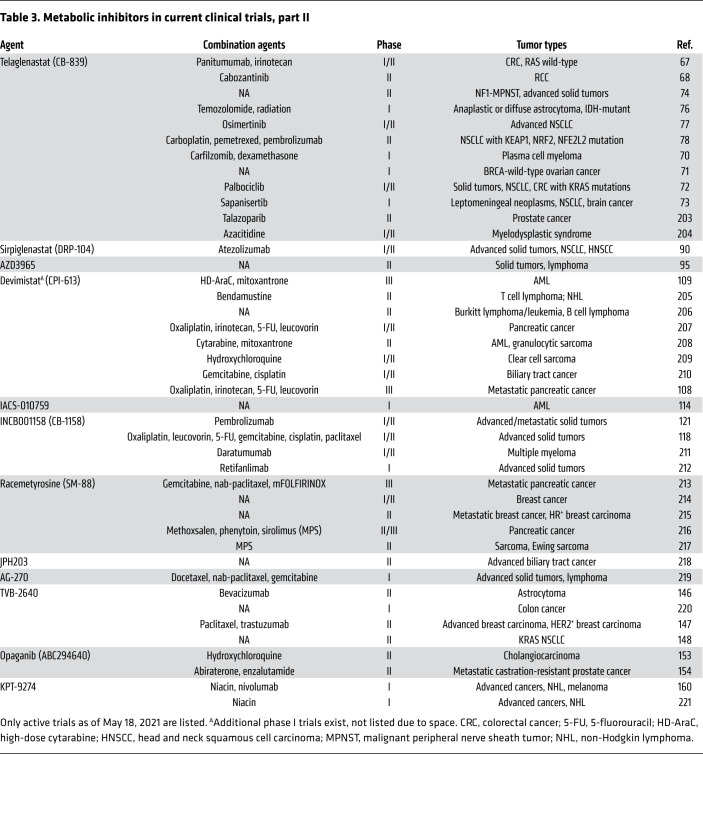
Metabolic inhibitors in current clinical trials, part II

**Table 2:**
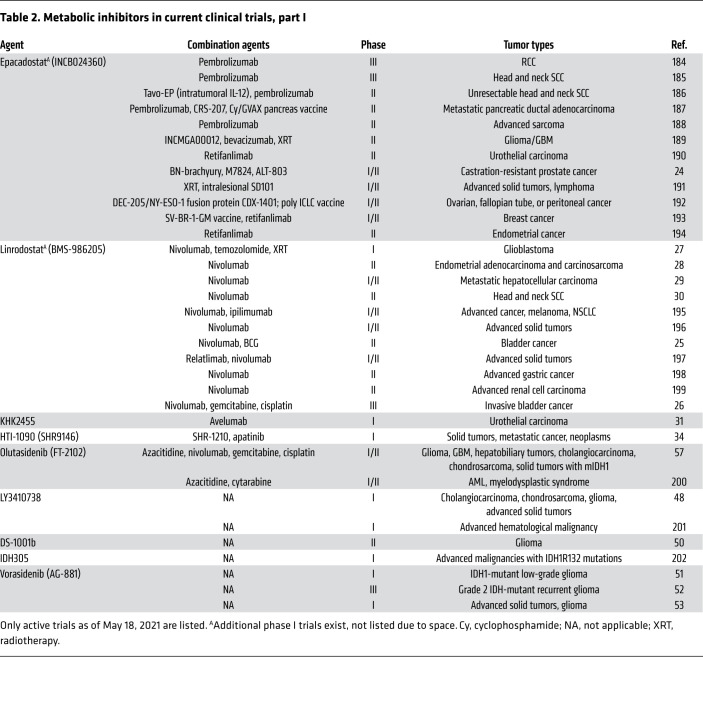
Metabolic inhibitors in current clinical trials, part I

**Table 1:**
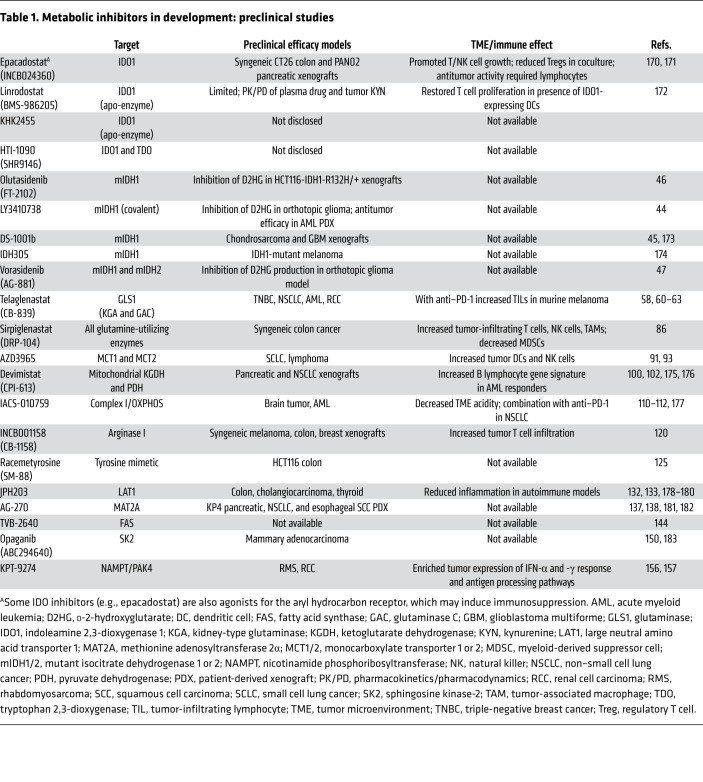
Metabolic inhibitors in development: preclinical studies

## Version 1 01/04/2022

Electronic publication
